# Effects of Volatile versus Total Intravenous Anesthesia on Occurrence of Myocardial Injury after Non-Cardiac Surgery

**DOI:** 10.3390/jcm8111999

**Published:** 2019-11-15

**Authors:** Ji-Hye Kwon, Jungchan Park, Seung-Hwa Lee, Ah-ran Oh, Jong-Hwan Lee, Jeong Jin Min

**Affiliations:** 1 Department of Anesthesiology and Pain Medicine, Samsung Medical Center, Sungkyunkwan University School of Medicine, Seoul 06351, Korea; kwonjh0828@hanmail.net (J.-H.K.); jc83.park@samsung.com (J.P.); ahran.oh@samsung.com (A.-r.O.); jonghwan75.lee@samsung.com (J.-H.L.); 2 Department of Cardiology, Samsung Medical Center, Sungkyunkwan University School of Medicine, Seoul 06351, Korea; shuaaa.lee@samsung.com

**Keywords:** myocardial injury after non-cardiac surgery, total intravenous anesthesia, volatile anesthesia, high-sensitivity cardiac troponin, remifentanil, acute kidney injury

## Abstract

The cardioprotective effects of volatile anesthetics versus total intravenous anesthesia (TIVA) are controversial, especially in patients undergoing non-cardiac surgery. Using current generation high-sensitivity cardiac troponin (hs-cTn), we aimed to evaluate the effect of anesthetics on the occurrence of myocardial injury after non-cardiac surgery (MINS). From February 2010 to December 2016, 3555 patients without preoperative hs-cTn elevation underwent non-cardiac surgery under general anesthesia. Patients were grouped according to anesthetic agent; 659 patients were classified into a propofol-remifentanil total intravenous anesthesia (TIVA) group, and 2896 patients were classified into a volatile group. To balance the use of remifentanil between groups, a balanced group (*n* = 1622) was generated with patients who received remifentanil infusion in the volatile group, and two separate comparisons were performed (TIVA vs. volatile and TIVA vs. balanced). The primary outcome was occurrence of MINS, defined as rise of hs-cTn I ≥ 0.04 ng/mL within postoperative 48 hours. The secondary outcomes were 30-day mortality, postoperative acute kidney injury (AKI), and adverse events during hospital stay (mortality, type I myocardial infarction (MI), and new-onset arrhythmia). In propensity-matched analyses, the occurrence of MINS was lower in the TIVA group compared to the volatile group (OR 0.642; 95% CI 0.450–0.914; *p* = 0.014). However, after balancing the use of remifentanil, there was no difference between groups in the risk of MINS (OR 0.832; 95% CI 0.554–1.251; *p*-value = 0.377). There were no significant associations between the two groups in type 1 MI, new-onset atrial fibrillation, in-hospital and 30-day mortality before and after balancing the use of remifentanil. However, the incidence of postoperative AKI was lower in the TIVA group (OR 0.362; 95% CI 0.194–0.675; *p*-value = 0.001). After balancing the use of remifentanil, volatile anesthesia and TIVA showed comparable effects on MINS in patients undergoing non-cardiac surgery without preoperative myocardial injury. Further studies are needed on the benefit of remifentanil infusion.

## 1. Introduction

Myocardial injury after non-cardiac surgery (MINS) is independently associated with an increased risk of mortality and major cardiac complications at 30 days and up to two years after surgery [1,2,3,4,5]. Current generation high-sensitivity cardiac troponin (hs-cTn) enables early detection of MINS; however, perioperative measures to prevent or minimize injury have not been determined [6]. 

Both volatile anesthetics and total intravenous anesthesia (TIVA) have cardioprotective effects through different mechanisms [7], and studies have extensively compared the protective effects of the two techniques [8]. Based on several clinical trials and meta-analyses, volatile anesthetics were identified as more cardioprotective than TIVA in patients undergoing cardiac surgery [8,9], but the result was not obvious in patients undergoing non-cardiac surgery [8,10]. Moreover, the most recent large, multicenter, randomized trial reported no mortality difference between the two techniques for up to one year, even in patients undergoing cardiac surgery [11].

Because MINS is mainly driven by mismatch of oxygen supply and demand, the use of other supportive drugs for hemodynamic stability or inherent risk factors should also be taken into account. In particular, remifentanil is reported to be cardioprotective by its own mechanism [12]. However, most previous studies did not address the effects of opioids or baseline troponin level before surgery.

In this study, we compared the occurrence of MINS between volatile anesthetics and propofol-remifentanil TIVA in patients undergoing non-cardiac surgery without preoperative myocardial injury. We also conducted a separate analysis after balancing the use of remifentanil between volatile anesthetic and TIVA groups.

## 2. Methods

### 2.1. Study Population and Data Collection

This study was approved by the Institutional Review Board of Samsung Medical Center (IRB No. 2018-12-002) and conducted in accordance with the principles of the Declaration of Helsinki. Considering the nature of a retrospective study and minimal risk to participants, the need for individual consent was waived by the IRB.

Anesthetic and postoperative management was performed according to institutional protocols based on current guidelines. Perioperative hs-cTn I measurement was not a routine practice but was selectively performed at the clinician’s discretion. A single highly sensitive immunoassay was performed using an automated analyzer (Advia Centaur XP, Siemens Healthcare Diagnostics, Erlangen, Germany). The lowest limit of detection was 0.006 ng/mL, and the normal limit was <0.04 ng/mL, according to the 99th percentile rule [13]. 

Our institution operates as a paperless hospital with an electronic medical record system that archives all patient medication information and laboratory findings. All data in this study were curated using “Clinical Data Warehouse Darwin-C,” an electronic system designed to search and retrieve de-identified medical records. From February 2010 to December 2016, all adult patients with measurement of hs-cTn I before and within 48 hours after non-cardiac surgery under general anesthesia at our institution were initially enrolled. Patients with preoperative myocardial injury or perioperative cardiopulmonary resuscitation were excluded. After finalizing patients for the study, independent researchers who were blinded to the perioperative medical data organized de-identified data including baseline characteristics and postoperative outcomes into a standardized form. 

**Figure 1:**
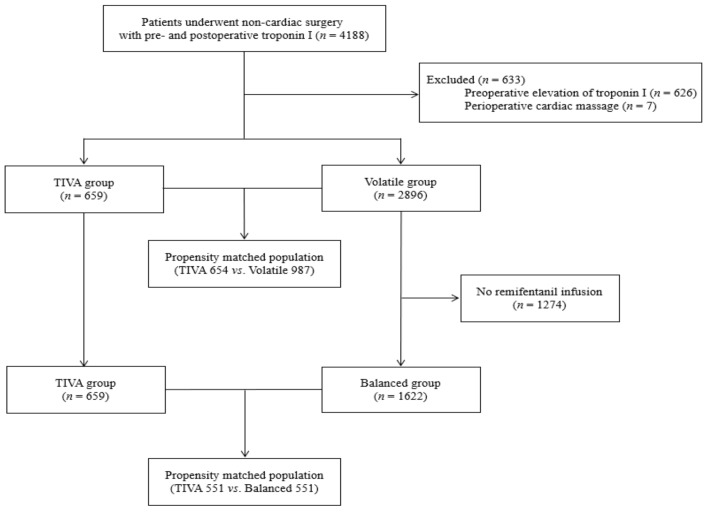
Flowchart of patients. TIVA indicates total intravenous anesthesia.

Patients were grouped according to anesthetic agent, which was chosen based on the attending anesthesiologist’s discretion; 661 patients were induced and maintained with propofol-remifentanil TIVA without use of a volatile agent (TIVA group), and 2901 patients were maintained with volatile anesthetic regardless of inducing agent (volatile group). In further analysis balancing the impact of continuously infused opioid, patients without remifentanil use were excluded from the volatile group, and patients who were maintained with volatile anesthetics in conjunction with remifentanil infusion were grouped into the balanced group (1622/2901) (Figure 1). Clinical outcomes of the TIVA group were compared to those of the balanced group and the volatile group.

### 2.2. Study Outcomes and Definitions

The primary outcome was MINS, defined as cardiac troponin elevation above the normal range (≥0.04 ng/mL) within postoperative 48 hours [5,14]. Secondary outcomes were 30-day mortality, postoperative acute kidney injury, and adverse events during hospital stay (mortality, type I myocardial infarction (MI), and new-onset arrhythmia). Type I MI was defined as evidence of coronary thrombus with symptoms or electrocardiographic changes compatible with ischemic etiology according to the Fourth Universal Definition of MI [14]. Postoperative AKI was defined based on the Kidney Disease Improving Global Outcomes (KDIGO) criteria using creatinine level. An absolute increase more than 0.3 mg/dl or a relative increase more than 50% from preoperative baseline level was definitive of AKI [15].

Previous medical history was based on preoperative evaluation records. Presence of hypertension was self-reported or based on prescription of anti-hypertensives or systolic blood pressure >140 mm Hg at rest. Diabetes mellitus was defined as a history of treatment, such as medication and lifestyle intervention, or diagnosis of type 1 or type 2 diabetes mellitus. History of stroke was defined as a history of neurological function loss caused by an ischemic or hemorrhagic event with residual symptoms at least 24 hours after onset. Chronic kidney disease was defined as any condition with gradual loss of kidney function with serum creatinine level consistently over 2.0 mg/dl or use of dialysis. Heart failure included either left ventricular dysfunction or congestive heart failure with preserved left ventricular function and was defined as a history of heart failure or use of loop diuretics accompanied by symptoms. Arrhythmia included any previously diagnosed alteration in heartbeat rhythm. Aortic disease was defined as acute or chronic pathologic lesion involving the thoracic or abdominal aorta. Operative risk was stratified according to 2014 European Society of Cardiology/Anesthesiology (ESC/ESA) guidelines [16]. 

### 2.3. Statistical Analysis

Continuous variables are described as mean (SD), and categorical variables are expressed as number (%). Baseline characteristics were compared between groups using the Mann–Whitney test or chi-square test for crude populations and a clustered linear model (continuous variables) or the Cochran–Mantel–Haenszel test (categorical variables) for matched populations. Matched populations were generated using propensity score matching to reduce selection bias and maximize study power while maintaining balance in confounding factors between groups. Variables for estimating propensity scores were preoperative (male, age, body mass index, current smoker, diabetes, hypertension, history of myocardial infarction, heart failure, valvular heart disease, peripheral arterial occlusive disease, carotid artery disease, aortic disease, pulmonary thromboembolism or deep venous thrombosis, arrhythmia, cerebrovascular disease, chronic kidney disease, dialysis, chronic liver disease, cancer, coronary artery disease, history of coronary artery bypass grafting and percutaneous coronary intervention, elevated C-reactive protein level, and medications) and intraoperative (emergent operation, operative risk, duration of operation, and intraoperative red blood cell transfusion) risk factors. The caliper width was 0.2 standard deviations of the logit-transformed propensity score. Reduction in the risk of outcome was compared using the logistic regression model. Odds ratio (OR) with 95% confidence interval (CI) was reported. We also performed a subgroup analysis to reveal hidden interaction with sex, chronic kidney disease, stroke, emergent operation, operation risk and intraoperative inotropic use. Multivariate logistic regression analysis was conducted to identify independent predictor of MINS. Variables included in analysis were anesthetic technique, sex, age, body mass index, diabetes, previous percutaneous coronary intervention, previous coronary artery bypass graft surgery, carotid arterial disease, history of stroke, chronic kidney disease, dialysis, heart failure, arrhythmia, valve disease, aortic disease, pulmonary thromboembolism or deep vein thrombosis, preoperative C-reactive protein (CRP) elevation, preoperative use of aspirin, beta blocker and clopidogrel, operation risk, emergent operation, operation duration, intraoperative requirement of inotropic agents or red blood cell transfusion.

All reported *P* values were two-sided, and *p* < 0.05 was considered significant. Statistical analyses were performed using SPSS 20.0 (IBM Corp., Chicago, IL) or R 3.5.2 (R Development Core Team, Vienna, Austria; http://www.R-project.org/).

## 3. Results

### 3.1. Patient Characteristics

The flowchart of patients is shown in Figure 1. A total of 4188 adult patients who underwent general anesthesia for noncardiac surgery with pre- and post-operative hs-cTn I measurements were initially enrolled. After excluding 626 patients with preoperative myocardial injury and 7 patients with perioperative cardiopulmonary resuscitation, a total of 3555 patients were left for analysis. Of the 3555 enrolled patients, 659 (18.5%) and 2896 (81.5%) were grouped into the TIVA and volatile groups, respectively (Table 1). After excluding 1274 patients without continuous infusion of remifentanil, 1622 (71.1%) patients were grouped into the balanced group and compared to 659 (28.9%) patients in the TIVA group (Table 2). Two separate propensity score matchings were performed to generate two population sets. After propensity score matching between the TIVA and volatile groups, 564 patients were grouped into the TIVA group, and 978 patients were grouped into the volatile group. In comparison between the TIVA and balanced groups, 551 patients were grouped into each group after propensity score matching (Figure 1). Standard mean differences <10% suggested well-balanced covariates in both sets of matched populations, and there were no significant differences in any variables between the compared study groups in the propensity score-matched cohort (Table 1; Table 2). Operation types according to operative risk in the entire population are described in Supplementary Table S1.

### 3.2. Anesthetic Techniques and MINS after Matching

After propensity score matching between the TIVA and volatile groups, the overall incidence of MINS was 13.0% (200/1542), with 10.1% (57/564) in the TIVA group and 15.0% (147/978) in the volatile group. The risk of MINS was significantly lower in the TIVA group in univariable and multivariable analyses (OR 0.636; 95% CI 0.459–0.880; *p*-value = 0.006 and OR 0.642; 95% CI 0.450–0.914; *p*-value = 0.014, Table 3). The median values of hs-cTn I for the patients with MINS were 0.080 (0.047–0.233) ng/mL in the TIVA group and 0.097 (0.059–0.442) ng/mL in the volatile group (*p*-value = 0.801).

In comparison between the TIVA and balanced groups, the overall incidence of MINS was 11.2% (123/1102). The incidence was 10.2% (56/551) in the TIVA group and 12.9% (71/551) in the balanced group. After balancing use of remifentanil, the risk of MINS was not significantly different in univariable and multivariable analyses (OR 0.765; 95% CI 0.527–1.110; *p*-value = 0.158 and OR 0.832; 95% CI 0.554–1.251; *p*-value = 0.377, Table 4). In this matched set of population, the median values of hs-cTn I for the patients with MINS were 0.082 (0.047–0.234) ng/mL in the TIVA group and 0.071 (0.052–0.190) ng/mL in the balanced group (*p*-value = 0.198). In subgroup analysis, no variables showed an interaction for MINS and the results are shown in Supplementary Figure S1.

### 3.3. Anesthetic Techniques and Other Secondary Outcomes after Matching

In the first analysis comparing TIVA and volatile groups, the incidence of postoperative AKI was lower in the TIVA group (OR 0.346; 95% CI 0.202–0.593; *p*-value < 0.001, Table 3). Significance remained in AKI stages 1 and 2, but not stage 3. Among patients with MINS, the incidence of AKI was 20.5%, and the incidence of type 1 MI was 3.0%. There were no significant associations between the two groups in type 1 MI, new-onset atrial fibrillation, in-hospital mortality, and 30-day mortality (Table 3).

In analysis between TIVA and balanced groups, the risk of postoperative AKI was still significantly lower in the TIVA group (OR 0.362; 95% CI 0.194–0.675; *p*-value = 0.001, Table 4). Among patients with MINS, the incidence of AKI was 21.1%, and the incidence of type 1 MI was 3.3%. There was no significant association between the two groups in type 1 MI, new-onset atrial fibrillation, in-hospital mortality, and 30-day mortality after matching (Table 4). In subgroup analysis, significant interaction between operation risk and the incidence of AKI was observed. Protective effect of TIVA was observed only in patients with intermediate-high operation risk (OR 0.39; 95% CI 0.21–0.71, *p*-value = 0.002 in intermediate-high risk group and OR 0.80; 95% CI 0.17–3.83, *p*-value = 0.78 in mild risk group, *p* for interaction = 0.001, Supplementary Figure S2).

### 3.4. Subanalysis of the Volatile Group: Volatile Only Group versus Balanced Group

The clinical outcomes of the volatile group were compared according to use of remifentanil and are presented in Supplementary Table S2. The overall incidence of MINS was 27.1% (785/2896), with 24.7% (315/1274) in the volatile only group and 15.5% (70/1622) in the balanced group. The risk of MINS was significantly lower in the balanced group in univariable analysis (OR 1.436; 95% CI 1.201–1.716; *p*-value < 0.0001). The incidence of postoperative AKI in all stages was lower in the balanced group (OR 1.803; 95% CI 1.447–2.248; *p*-value < 0.0001). The incidence of in-hospital mortality and 30-day death was also lower in the balanced group than in the gas only group (OR 1.070; 95% CI 2.638–6.279; *p*-value < 0.0001 and OR 2.338; 95% CI 1.517–3.604; *p*-value < 0.0001). There was no significant association between the two groups in type 1 MI, new-onset arrhythmia, and new-onset atrial fibrillation (Supplemental Table S2).

### 3.5. Predictors of MINS

Variables associated with MINS are shown in Table 5. In univariate analysis, anesthetic technique, body mass index, diabetes, previous percutaneous coronary intervention, history of stroke, chronic kidney disease, dialysis, heart failure, arrhythmia, valve disease, aortic disease, preoperative CRP elevation, previous use of beta blocker and clopidogrel, operation risk, emergent operation, operation duration, intraoperative inotropic requirement and red blood cell transfusion were associated with MINS. In multivariate analysis, patients who underwent TIVA had a lesser risk of MINS (OR 0.62; 95% CI 0.46–0.84, *p*-value = 0.002) than those who underwent volatile anesthesia. In addition, sex, age, body mass index, previous coronary artery bypass graft surgery, history of stroke, chronic kidney disease, valve disease, operation risk, emergent operation, operation duration, intraoperative inotropic requirement and red blood cell transfusion were also related with the incidence of MINS. 

## 4. Discussion

### 4.1. Summary of Results

The present study compared the effects of volatile anesthetics versus propofol-remifentanil TIVA on the occurrence of MINS and other adverse outcomes in patients undergoing non-cardiac surgery. After balancing use of remifentanil, the occurrence of MINS was comparable between volatile anesthetic and TIVA groups. Moreover, other major postoperative adverse outcomes did not differ significantly between the two groups, except for AKI. The incidence of postoperative AKI was lower in the TIVA group.

### 4.2. Current Evidence for Volatile Anesthetics vs. TIVA in Non-Cardiac Surgery

Because both general anesthesia techniques have cardioprotective effects, comparison of the cardioprotective effects of the two anesthetic techniques is a long-standing subject of debate. Volatile anesthetics are cardioprotective via myocardial preconditioning and have been shown to reduce myocardial infarct size in models and reduce postoperative mortality compared to TIVA in patients undergoing cardiac surgery [7,17]. However, propofol has been shown as well to be organ-protective via anti-inflammatory, immune-modulatory, and antioxidant properties [18,19,20]. Moreover, the recently published international MortaliY in caRdIAc surgery ranDomized (MYRIAD) clinical trial reported comparable outcomes between the two techniques, further confusing the conclusions in patients undergoing cardiac surgery [11]. 

In non-cardiac surgery, cardioprotective effects associated with volatile anesthetics found in cardiac surgery were not obvious [8,10]. Therefore, current guidelines recommend use of either volatile anesthetics or TIVA for patients undergoing non-cardiac surgery, and the choice of anesthetic agent is determined by factors other than prevention of myocardial ischemia [16,21]. However, ischemic symptoms are likely to be masked under sedatives or surgical pain in the postoperative period [14], and recent evidences in non-cardiac surgery indicated that clinically silent elevation of cardiac troponin without ischemic symptom was still associated with increased risk of postoperative mortality [1,2,3,4]. Therefore, more research on the choice of anesthetics in non-cardiac surgery is needed [22,23]. In this study, instead of reporting the incidence of Type 2 myocardial infarction, which could be inaccurate, we evaluated whether cardioprotective effects of anesthetics show difference regarding myocardial injury, solely defined by cardiac troponin elevation. In addition, we compared the incidence of Type 1 myocardial infarction which can be angiographically proven. 

### 4.3. Possible Implications of Our Findings

Unlike previous studies, we considered remifentanil use in conjunction with a volatile agent or propofol during general anesthesia, and we only enrolled patients with normal baseline serum troponin to exclude myocardial injury that might have existed before the operation. In the majority of previous studies, preoperative troponin was identified only in part of the study population and use of intraoperative opioid was not addressed when comparing the effects of the two anesthetic techniques.

Without considering the effect of remifentanil infusion in our analysis, use of volatile anesthetics was associated with higher incidence of MINS. After balancing remifentanil by excluding patients without remifentanil infusion in the comparison between TIVA and balanced groups, this association was no longer significant, suggesting a benefit of remifentanil use. This benefit could be because MINS is mostly related to type 2 MI driven by oxygen supply/demand mismatch [14]. Stimulation of the sympathetic nervous system precipitates cardiovascular events during the perioperative period [6]. Intraoperative use of remifentanil effectively provides adequate protection against this stimuli with rapid onset and offset of action irrespective of its administration duration [24,25,26]. A meta-analysis reported that remifentanil not only facilitates early recovery with shorter time required for mechanical ventilation and length of hospital stay, but also reduces cardiac troponin release after cardiac surgery [27]. Preconditioning effects on the heart and drug interactions with volatile anesthetics and maintenance of Zinc homeostasis leading to attenuation of endoplasmic reticulum stress and myocardial ischemia/reperfusion injury could also be related to the benefits of remifentanil [7,12,27]. In comparison within the volatile group (Supplementary Table S2), patients with remifentanil use showed lower incidence of MINS, and the protective effect was significant in the univariate model. However, the benefit of remifentanil use is beyond the scope of this study and requires further investigation.

### 4.4. Postoperative AKI

Interestingly, the use of TIVA consistently showed a protective effect against postoperative AKI compared to the use of volatile agent regardless of remifentanil use. The reno-protective effects of propofol have been shown in animal experiments and have also been reported in clinical settings to be superior to those of volatile anesthetics. Considering that the putative pathophysiology of postoperative AKI includes inflammation, oxidative stress, cellular necrosis, and apoptosis caused by possible ischemia/reperfusion injury, the reno-protective mechanisms of propofol seemed to be mainly attributed to anti-inflammatory, anti-oxidative, anti-necrotic and anti-apoptotic abilities to the kidney through different mechanisms [28]. Previous studies have indicated that propofol had immunomodulatory effects by regulating microRNA signaling pathway and reduced the inflammatory cytokines in the kidney [20,29]. Propofol also had antioxidant abilities by lowering the formation of oxidative stress markers and more preserving superoxide dismutase levels [30,31]. In addition, because propofol is a lipid emulsion, propofol has shown kidney protective effects against ischemia/reperfusion injury through efficient membrane targeted and cytoprotective effects by preventing uncontrolled opening of the mitochondrial permeability transition pore after ischemia, which leads to the release of pro-apoptotic factors and necrotic cell death [28].

Another aspect to consider is that renal injury can cause extra-cardiac hs-cTn elevation [4,32]. Therefore, it is possible that the cardioprotective effect of volatile anesthetics in non-cardiac surgery might be attenuated to an extent that the reno-protective effect of propofol in TIVA can compensate. However, whether the use of TIVA clearly benefits the population at high risk for postoperative AKI remains uncertain after this study. The subgroup analysis showed that this protective effect of TIVA was significant irrespective of chronic kidney disease but limited to intermediate-high risk operation.

### 4.5. Other Things to Consider for Drug Selection in Non-Cardiac Surgery

There are several things to consider other than cardio-protection when choosing anesthetic agents in patients undergoing non-cardiac surgery. Several clinical studies and recent meta-analyses have reported that the use of propofol-based TIVA may be associated with better recurrence-free and overall survival in patients undergoing cancer surgery [33,34,35]. As stated in the current guidelines for non-cardiac surgery, it is reasonable to choose anesthetic drugs by considering factors other than prevention of myocardial ischemia, such as organs at risk or long-term cancer recurrence. However, further research is needed for more detailed recommendations in specific patient subgroups.

### 4.6. Study Limitations

This study has several limitations. First, this was not a prospective randomized study; therefore, we could not exclude the possibility of bias from hidden or unobserved variables despite efforts to include all established contributors to occurrence of MINS. We retained all types of non-cardiac surgeries, so heterogeneity in operative burden and inherent patient risks might have also influenced the results. Second, perioperative hs-cTn was not routinely measured in all patients, so enrolling only patients with pre- and postoperative hs-cTn could have resulted in selection bias. Third, the use of opioid other than remifentanil or different induction agents in the volatile group was not considered. In addition, because propofol was used in conjunction with remifentanil in every case of the TIVA group, the effect of the individual agent (propofol or volatile agent) could not be discussed. However, considering that remifentanil is not used alone in general anesthesia, our data are more likely to reflect real-world data. Finally, an association with actual adverse events during follow-up was not shown in this study. Despite these limitations, this study evaluated the effects of anesthetic agents on MINS in all types of non-cardiac surgery and has clinical impacts on the daily practice of nearly all anesthesiologists.

## 5. Conclusions

After balancing the use of remifentanil, volatile anesthetics and TIVA showed comparable effects on the occurrence of MINS in patients undergoing non-cardiac surgery without preoperative myocardial injury. Further studies are needed regarding the effects of anesthetic techniques in different patient subgroups or the benefit of remifentanil infusion in patients undergoing non-cardiac surgery.

## Figures and Tables

**Table 1:** Balance in clinical characteristics between the TIVA and volatile groups, before and after matching.

	Before Matching				After Matching			
	TIVA (*n* = 659)	Volatile (*n* = 2896)	*P*	SMD	TIVA (*n* = 564)	Volatile (*n* = 978)	*P*	SMD
Male sex	299 (45.4)	2014 (69.5)	<0.001	0.504	289 (51.2)	542 (55.4)	0.125	0.084
Age, years	63.02 (11.94)	64.89 (± 12.71)	<0.001	0.152	63.50 (11.78)	63.85 (13.21)	0.595	0.028
Smoking	52 (7.9)	413 (14.3)	<0.001	0.204	50 (8.9)	99 (10.1)	0.474	0.043
BMI	24.33 (3.76)	23.68 (3.66)	<0.001	−0.175	24.14 (3.69)	24.03 (3.79)	0.586	0.029
Comorbidities								
Hypertension	321 (48.7)	1538 (53.1)	0.046	0.088	278 (49.3)	492 (50.3)	0.740	0.020
Diabetes	147 (22.3)	803 (27.7)	0.005	0.125	138 (24.5)	251 (25.7)	0.645	0.028
Old MI	28 (4.2)	151 (5.2)	0.355	0.045	27 (4.8)	56 (5.7)	0.503	0.042
Previous PCI	48 (7.3)	340 (11.7)	0.001	0.152	47 (8.3)	89 (9.1)	0.676	0.027
Previous CABG	15 (2.3)	147 (5.1)	0.003	0.149	15 (2.7)	39 (4.0)	0.221	0.074
PAOD	15 (2.3)	327 (11.3)	<0.001	0.364	15 (2.7)	37 (3.8)	0.303	0.064
Carotid arterial disease	56 (8.5)	711 (24.6)	<0.001	0.237	97 (17.2)	188 (19.2)	0.358	0.052
COPD	69 (10.5)	343 (11.8)	0.354	0.044	62 (11.0)	110 (11.2)	0.945	0.008
History of stroke	78 (11.8)	419 (14.5)	0.090	0.078	73 (12.9)	139 (14.2)	0.535	0.037
Chronic kidney disease	29 (4.4)	236 (8.1)	0.001	0.155	28 (5.0)	56 (5.7)	0.604	0.034
Dialysis	9 (1.4)	70 (2.4)	0.132	0.077	9 (1.6)	20 (2.0)	0.667	0.034
Cancer	119 (18.1)	672 (23.2)	0.005	0.127	112 (19.9)	212 (21.7)	0.436	0.045
Heart failure EF.	9 (1.4)	56 (1.9)	0.412	0.045	9 (1.6)	17 (1.7)	0.997	0.011
Arrythmia	48 (7.3)	264 (9.1)	0.154	0.067	47 (8.3)	81 (8.3)	1.000	−0.002
Valve disease	18 (2.7)	83 (2.9)	0.954	0.008	18 (3.2)	28 (2.9)	0.834	−0.019
Aortic disease	8 (1.2)	237 (8.2)	<0.001	0.334	8 (1.4)	17 (1.7)	0.787	0.026
PTE DVT	12 (1.8)	52 (1.8)	1.000	−0.002	10 (1.8)	17 (1.7)	1.000	−0.003
Preop. CRP elevation	160 (24.3)	1262 (43.6)	<0.001	0.416	156 (27.7)	297 (30.4)	0.286	0.060
Medication								
ACEi_ARB	165 (25.0)	825 (28.5)	0.083	0.078	148 (26.2)	255 (26.1)	0.990	−0.004
Aspirin	141 (21.4)	831 (28.7)	<0.001	0.169	131 (23.2)	241 (24.6)	0.573	0.033
BB	82 (12.4)	578 (20.0)	<0.001	0.205	76 (13.5)	147 (15.0)	0.446	0.045
CCB	165 (25.0)	772 (26.7)	0.422	0.037	145 (25.7)	251 (25.7)	1.000	−0.001
clopidogrel	55 (8.3)	402 (13.9)	<0.001	0.177	54 (9.6)	104 (10.6)	0.566	0.035
Statin	152 (23.1)	806 (27.8)	0.015	0.110	136 (24.1)	245 (25.1)	0.726	0.022
Intraoperative parameter								
OP risk			<0.001				0.086	
Low	38 (5.8)	219 (7.6)		0.072	38 (6.7)	73 (7.5)		0.028
Intermediate	611 (92.7)	2039 (70.4)		−0.601	516 (91.5)	869 (88.9)		−0.089
High	10 (1.5)	638 (22.0)		0.671	10 (1.8)	36 (3.7)		0.117
Emergent operation	87 (13.2)	695 (24.0)	<0.001	0.280	84 (14.9)	168 (17.2)	0.273	0.062
OP duration	211.28 (124.81)	208.92 (145.48)	0.671	−0.017	205.30 (118.04)	195.11 (134.72)	0.122	0.080
inotropic requirement	76 (11.5)	940 (32.5)	<0.001	0.522	75 (13.3)	161 (16.5)	0.112	0.089
RBC transfusion	0.87 (0.59)	0.75 (0.74)	<0.001	−0.176	0.83 (0.59)	0.79 (0.82)	0.266	0.056

Values are *n* (%) or mean ± SD. TIVA indicates total intravenous anesthesia. Abbreviation: BMI, body mass index; MI, myocardial infarction; PCI, percutaneous coronary intervention; CABG, coronary artery bypass grafting; PAOD, peripheral artery occlusion disease; COPD, chronic obstructive pulmonary disease; PTE, pulmonary thromboembolism; DVT, deep vein thrombosis; CRP, C-reactive protein; ACEi, angiotensin-converting enzyme inhibitor; ARB, angiotensin 2 receptor blocker; BB, beta blocker; CCB, calcium channel blocker; OP, operation; RBC, red blood cell; SMD, standard mean difference. For continuous variables, Wilcoxon rank sum test, paired *t* test or Wilcoxon signed rank test was used. For categorical variables, x or McNemar test was used

**Table 2:** Balance in clinical characteristics between the two groups before and after matching.

	Before Matching				After Matching			
	TIVA (*n* = 659)	Balanced (*n* = 1622)	*P*	SMD	TIVA (*n* = 551)	Balanced (*n* = 551)	*P*	SMD
Male sex	299 (45.4)	1148 (70.8)	<0.001	−0.533	288 (52.3)	290 (52.6)	0.952	−0.007
Age, years	63.02 (11.94)	65.61 (12.45)	<0.001	0.213	63.34 (11.61)	64.20 (13.43)	0.257	−0.068
Smoking	52 (7.9)	249 (15.4)	<0.001	−0.234	51 (9.3)	49 (8.9)	0.916	0.013
BMI	24.33 (3.76)	23.78 (3.61)	0.001	0.149	24.16 (3.67)	24.17 (3.76)	0.958	−0.003
Comorbidities								
Hypertension	321 (48.7)	949 (58.5)	<0.001	−0.197	277 (50.3)	284 (51.5)	0.718	−0.025
Diabetes	147 (22.3)	492 (30.3)	<0.001	−0.183	135 (24.5)	138 (25.0)	0.889	−0.013
Old MI	28 (4.2)	92 (5.7)	0.202	−0.066	28 (5.1)	30 (5.4)	0.893	−0.016
Previous PCI	48 (7.3)	208 (12.8)	<0.001	−0.185	48 (8.7)	44 (8.0)	0.744	0.026
Previous CABG	15 (2.3)	101 (6.2)	<0.001	−0.197	15 (2.7)	16 (2.9)	1.000	−0.011
PAOD	15 (2.3)	238 (14.7)	<0.001	−0.457	15 (2.7)	16 (2.9)	1.000	−0.011
Carotid arterial disease	56 (8.5)	230 (14.2)	<0.001	−0.18	56 (10.2)	53 (9.6)	0.840	0.018
COPD	69 (10.5)	210 (12.9)	0.117	−0.077	65 (11.8)	68 (12.3)	0.853	−0.017
History of stroke	78 (11.8)	255 (15.7)	0.021	−0.113	70 (12.7)	82 (14.9)	0.337	−0.063
Chronic kidney disease	29 (4.4)	121 (7.5)	0.010	−0.13	27 (4.9)	33 (6.0)	0.507	−0.048
Dialysis	9 (1.4)	29 (1.8)	0.594	−0.034	9 (1.6)	11 (2.0)	0.821	−0.027
Cancer	119 (18.1)	303 (18.7)	0.773	−0.016	108 (19.6)	115 (20.9)	0.653	−0.032
Heart failure EF.	9 (1.4)	36 (2.2)	0.245	−0.064	9 (1.6)	11 (2.0)	0.821	−0.027
Arrythmia	48 (7.3)	154 (9.5)	0.109	−0.08	47 (8.5)	58 (10.5)	0.305	−0.068
Valve disease	18 (2.7)	51 (3.1)	0.699	−0.024	18 (3.3)	19 (3.4)	1.000	−0.01
Aortic disease	8 (1.2)	194 (12.0)	<0.001	−0.444	8 (1.5)	12 (2.2)	0.498	−0.054
PTE DVT	12 (1.8)	29 (1.8)	1.000	−0.002	10 (1.8)	13 (2.4)	0.673	−0.038
Medication								
ACEi_ARB	165 (25.0)	514 (31.7)	0.002	−0.148	144 (26.1)	136 (24.7)	0.628	0.033
Aspirin	141 (21.4)	535 (33.0)	<0.001	−0.263	131 (23.8)	125 (22.7)	0.721	0.026
BB	82 (12.4)	349 (21.5)	<0.001	−0.243	80 (14.5)	84 (15.2)	0.800	−0.02
CCB	165 (25.0)	484 (29.8)	0.024	−0.108	148 (26.9)	141 (25.6)	0.681	0.029
clopidogrel	55 (8.3)	260 (16.0)	<0.001	−0.237	54 (9.8)	51 (9.3)	0.837	0.019
Statin	152 (23.1)	533 (32.9)	<0.001	−0.22	140 (25.4)	140 (25.4)	1.000	0
Preop. CRP elevation	160 (24.3)	618 (38.1)	<0.001	−0.302	155 (28.1)	178 (32.3)	0.149	−0.091
Intraoperative parameter								
OP risk			<0.001				0.659	
Low	38 (5.8)	90 (5.5)		0.009	36 (6.5)	39 (7.1)		−0.022
Intermediate	611 (92.7)	1171 (72.2)		0.56	505 (91.7)	498 (90.4)		0.044
High	10 (1.5)	361 (22.3)		−0.676	10 (1.8)	14 (2.5)		−0.05
Emergent operation	87 (13.2)	276 (17.0)	0.028	−0.107	83 (15.1)	89 (16.2)	0.678	−0.03
OP duration	211.28 (124.81)	203.18 (117.68)	0.143	0.067	205.19 (120.97)	198.91 (125.69)	0.398	−0.051
inotropic requirement	76 (11.5)	491 (30.3)	<0.001	−0.474	75 (13.6)	91 (16.5)	0.206	−0.081
RBC transfusion	0.87 (0.59)	0.74 (0.61)	<0.001	0.214	0.81 (0.55)	0.81 (0.62)	0.959	−0.003

Values are *n* (%) or mean ± SD. TIVA indicates total intravenous anesthesia. Abbreviation: BMI, body mass index; MI, myocardial infarction; PCI, percutaneous coronary intervention; CABG, coronary artery bypass grafting; PAOD, peripheral artery occlusion disease; COPD, chronic obstructive pulmonary disease; PTE, pulmonary thromboembolism; DVT, deep vein thrombosis; CRP, C-reactive protein; ACEi, angiotensin-converting enzyme inhibitor; ARB, angiotensin 2 receptor blocker; BB, beta blocker; CCB, calcium channel blocker; OP, operation; RBC, red blood cell; SMD, standard mean difference. For continuous variables, Wilcoxon rank sum test, paired *t* test or Wilcoxon signed rank test was used. For categorical variables, x or McNemar test was used

**Table 3:** Clinical outcomes comparing TIVA versus volatile groups in matched cohort.

	TIVA (*n* = 564)	Volatile (*n* = 97 )	Univariable Analysis		Multivariable Analysis	
			Unadjusted OR (95% CI)	*P*-Value	Adjusted OR (95% CI)	*P*-Value
Primary Outcome						
MINS	57 (10.1)	147 (15.0)	0.636 (0.459–0.880)	0.006	0.642 (0.450–0.914)	0.014
Secondary Outcomes						
30-day mortality	10 (1.77)	32 (3.27)	0.534 (0.260–1.094)	0.086	0.617 (0.294–1.293)	0.201
AKI, all stage	18 (3.19)	86 (8.79)	0.342 (0.203–0.575)	<0.001	0.346 (0.202–0.593)	0.0001
AKI 1	16 (2.83)	67 (6.85)	0.397 (0.228–0.692)	0.001	0.395 (0.223–0.701)	0.002
AKI 2	1 (0.17)	15 (1.53)	0.114 (0.015–0.866)	0.036	0.108 (0.013–0.928)	0.043
AKI 3	1 (0.17)	4 (0.41)	0.432 (0.048–3.879)	0.454	0.301 (0.027–3.385)	0.331
In-hospital events						
Mortality	13 (2.31)	32 (3.27)	0.697 (0.363–1.340)	0.281	0.955 (0.472–1.932)	0.897
Myocardial infarction	3 (0.53)	8 (0.81)	0.648 (0.171–2.454)	0.523	0.699 (0.182–2.686)	0.602
New arrythmia	14 (2.48)	30 (3.06)	0.804 (0.423–1.530)	0.507	0.783 (0.404–1.517)	0.467
New atrial fibrillation	11 (1.95)	31 (3.16)	0.161 (0.303–1.218)	0.608	0.581 (0.284–1.189)	0.137

Values are *n* (%). TIVA indicates total intravenous anesthesia and AKI indicates acute kidney injury Abbreviation: OR, odds ratio. MINS indicates myocardial injury after non-cardiac surgery.

**Table 4:** Clinical outcomes comparing TIVA versus balanced groups in matched cohort.

	TIVA (*n* = 551)	Balanced (*n* = 551)	Univariable Analysis		Multivariable Analysis	
			Unadjusted OR (95% CI)	*P*-Value	Adjusted OR (95% CI)	*P*-Value
Primary Outcome						
MINS	56 (10.2)	71 (12.9)	0.765 (0.527–1.110)	0.158	0.832 (0.554–1.251)	0.377
Secondary Outcomes						
30-day mortality	10 (0.22)	16 (0.35)	0.618 (0.278–1.374)	0.238	0.597 (0.256–1.395)	0.233
AKI, all stage	18 (3.99)	41 (9.11)	0.420 (0.238–0.741)	0.003	0.362 (0.194–0.675)	0.001
AKI 1	15 (3.33)	36 (7.98)	0.400 (0.217–0.740)	0.003	0.358 (0.184–0.698)	0.003
AKI 2	2 (0.43)	4 (0.88)	0.498 (0.091–2.731)	0.422	0.238 (0.021–2.668)	0.244
AKI 3	1 (0.22)	1 (0.22)	1.000 (0.062–16.01)	1.001	1.201 (0.048–29.98)	0.911
In-hospital events						
Mortality	14 (3.11)	13 (2.88)	1.079 (0.502–2.317)	0.846	1.315 (0.577–2.995)	0.515
Myocardial infarction	3 (0.66)	3 (0.66)	1.000 (0.201–4.976)	1.001	1.105 (0.216–5.644)	0.905
New arrythmia	11 (2.43)	14 (3.11)	0.781 (0.352–1.737)	0.545	0.832 (0.371–1.864)	0.655
New atrial fibrillation	8 (1.77)	18 (3.99)	0.436 (0.188–1.012)	0.053	0.486 (0.202–1.168)	0.107

Values are *n* (%). TIVA indicates total intravenous anesthesia and AKI indicates acute kidney injury. Abbreviation: OR, odds ratio.

**Table 5:** Variables associated with myocardial injury after non-cardiac surgery (MINS).

	Univariate Analysis		Multivariate Analysis	
	Unadjusted OR (95% CI)	*P*-Value	Adjusted OR (95% CI)	*P*-Value
**Anesthetic technique**	0.38 (0.29–0.50)	<0.001	0.62 (0.46–0.84)	0.002
Male sex	1.15 (0.97–1.37)	0.111	0.78 (0.64–0.95)	0.015
Age, years	1.01 (1.00–1.01)	0.05	1.02 (1.01–1.03)	<0.001
Smoking	0.91 (0.71–1.17)	0.454		
BMI	0.97 (0.95–0.99)	0.013	0.97 (0.95–1.00)	0.025
**Comorbidities**				
Hypertension	1.11 (0.94–1.31)	0.209		
Diabetes	1.21 (1.01–1.45)	0.039	1.06 (0.85–1.31)	0.609
Old MI	1.02 (0.71–1.49)	0.879		
Previous PCI	1.45 (1.14–1.85)	0.003	1.44 (0.98–2.12)	0.064
Previous CABG	2.11 (1.51–2.96)	<0.001	2.05 (1.32–3.29)	0.001
PAOD	1.00 (0.76–1.32)	0.981		
Carotid arterial disease	1.03 (0.80–1.33)	0.821	1.08 (0.78–1.50)	0.636
COPD	1.08 (0.84–1.39)	0.542		
History of stroke	1.39 (1.12–1.74)	0.003	1.40 (1.08–1.82)	0.011
Chronic kidney disease	3.65 (2.82–4.73)	<0.001	3.55 (2.54–5.00)	<0.001
Dialysis	2.76 (1.75–4.37)	<0.001	0.77 (0.42–1.41)	0.401
Cancer	1.02 (0.94–1.24)	0.839		
Heart failure	2.35 (1.41–3.92)	0.001	1.79 (0.94–3.30)	0.065
Arrythmia	1.35 (1.03–1.77)	0.028	1.16 (0.84–1.59)	0.375
Valve disease	2.05 (1.65–3.13)	0.001	2.28 (1.43–3.64)	0.001
Aortic disease	2.24 (1.70–2.96)	<0.001	0.98 (0.71–1.37)	0.915
PTE DVT	0.56 (0.27–1.18)	0.126	0.50 (0.22–1.15)	0.103
Preop. CRP elevation	0.71 (0.60–0.84)	<0.001	1.14 (0.94–1.39)	0.194
**Medication**				
ACEi_ARB	0.97 (0.81–1.17)	0.764		
Aspirin	1.19 (1.00–1.43)	0.056	1.20 (0.95–1.52)	0.131
BB	1.54 (1.27–1.88)	<0.001	1.10 (0.87–1.40)	1.103
CCB	1.06 (0.88–1.27)	0.538		
Clopidogrel	1.31 (1.04–1.66)	0.021	1.11 (0.82–1.51)	0.497
Statin	1.05 (0.87–1.26)	0.622		
**Intraoperative parameter**				
Operation risk		<0.001		0.021
Low				
Intermediate	0.91 (0.65–1.27)		0.75 (0.52–1.09)	
High	2.16 (1.52–3.08)		1.05 (0.68–1.60)	
Emergent operation	1.69 (1.41–2.04)	<0.001	2.11 (1.68–2.65)	<0.001
Operation duration	1.00 (1.00–1.00)	<0.002	1.00 (1.00–1.00)	<0.001
Inotropic requirement	5.08 (1.27–6.04)	<0.001	3.38 (2.78–4.12)	<0.001
RBC transfusion	1.82 (1.61–2.05)	<0.001	1.38 (1.21–1.58)	<0.001

## Supplementary Materials

The following are available online at https://www.mdpi.com/2077-0383/8/11/1999/s1, Figure S1. Subgroup analysis for MINS, Figure S2. Subgroup analysis for AKI, Table S1: Operation types according to operative risk in entire population, Table S2: Clinical outcomes comparing volatile anesthetics only group versus Balanced group.
