# Supplementary material for: Effects of Volatile versus Total Intravenous Anesthesia on Occurrence of Myocardial Injury after Non-Cardiac Surgery
Source: J Clin Med. 2019 Nov 15;8(11):1999. doi: 10.3390/jcm8111999 (PMC6912591; doi:10.3390/jcm8111999)
Supplement: Supplementary file 1 [file jcm-08-01999-s001.pdf]

Supplementary Table 1

Operation types according to operative risk in entire population.

Op risk	Operation type	Number (%)
Low	Superficial surgery	23 (0.6)
	Breast	52 (1.5)
	Dental	12 (0.3)
	Endocrine: thyoid	49 (1.4)
	Eye	3 (0.1)
	Reconstructive	7 (0.2)
	Carotid asymptomatic (CEA or CAS)	4 (0.1)
	Gynaecology:minor	7 (0.2)
	Orphopaedic:minor (meniscectomy)	72 (2.0)
	Urological:minor (TURP)	28 (0.8)
Intermediate	Intraperitoneal: splenectomy, hiatal hernia repair, cholecystectomy	786 (22.1)
	Carotid symptomatic (CEA or CAS)	352 (9.9)
	Peripheral arterial angioplasty	484 (13.6)
	Endovascular Anurysm repair	32 (0.9)
	Head and neck surgery	219 (6.2)
	Neurological or orphopaedic:major (hip and spine surgery)	668 (18.8)
	Urological or gynaecological:maajor	15 (0.4)
	Renal transplant	46 (1.3)
	Intra-thoracic: non-major	48 (1.4)
High	Aortic and major vascular surgery	128 (3.6)
	Open lower limb revascularization or amputation or thromboembolectomy	117 (3.3)
	Duodeno-pancreatic surgery	103 (2.9)
	Liver resection, bile duct surgery	69 (1.9)
	Oesophagectomy	19 (0.5)
	Reparir of perforated bowel	75 (2.1)
	Adrenal resection	8 (0.2)
	Total cystectomy	4 (0.1)
	Pneumonectomy	21 (0.6)
	Pulmonary or liver transplant	104 (2.9)

Values are *n* (%). CEA indicates carotid endarterectomy. Abbreviation: CAS = carotid artery stenting; TURP = trans-urethral resection of the prostate.

Supplementary Table 2

Clinical outcomes comparing volatile anesthetics only group versus Balanced group.

	**Volatiles only (*N* = 1274)**	**Balanced (*N* = 1622)**	**Univariable analysis**	
			**Unadjusted OR (95% CI)**	***P*-value**
**Primary Outcome**				
MINS	315(24.7)	70 (15.52)	1.436 (1.201-1716)	<0.0001
Secondary Outcomes				
30-day mortality	59 (4.6)	33 (2.0)	2.338 (1.517-3.604)	<0.0001
AKI, all stage	210 (16.5)	160 (9.9)	1.803 (1.447-2.248)	<0.0001
AKI 1	156 (12.2)	136 (8.4)	1.525 (1.196-1.943)	0.001
AKI 2	41 (3.2)	21 (1.3)	2.535 (1.490-4.312)	0.001
AKI 3	13 (1.0)	3 (0.2)	5.564 (1.582-19.566)	0.007
In-hospital events				
Mortality	85 (6.7)	28 (1.7)	1.070 (2.638-6.279)	<0.0001
Myocardial infarction	7 (0.5)	14 (0.9)	0.635 (0.255-1.577)	0.327
New arrythmia	52 (4.1)	51 (3.1)	1.311 (0.884-1.943)	0.178
New atrial fibrillation	39 (3.1)	53 (3.3)	0.935 (0.614-1.423)	0.753

Values are *n* (%). MINS indicates myocardial injury after non-cardiac surgery. Abbreviation: AKI, acute kidney injury; OR, odds ratio.

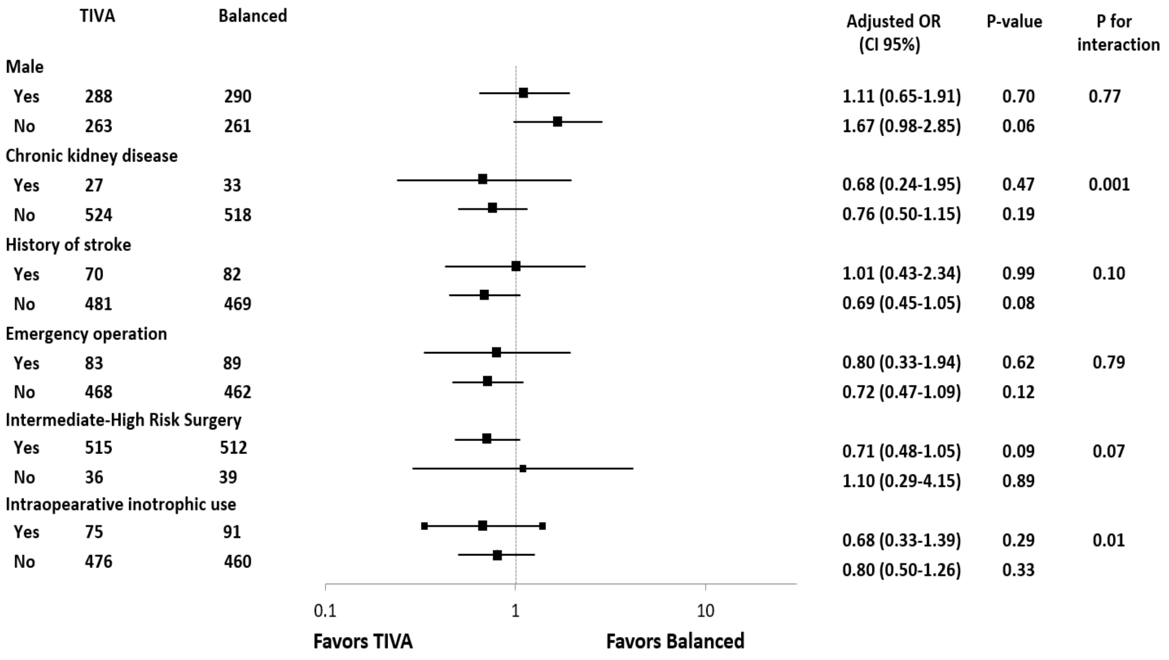

Figure 1. Subgroup analysis for MINS.

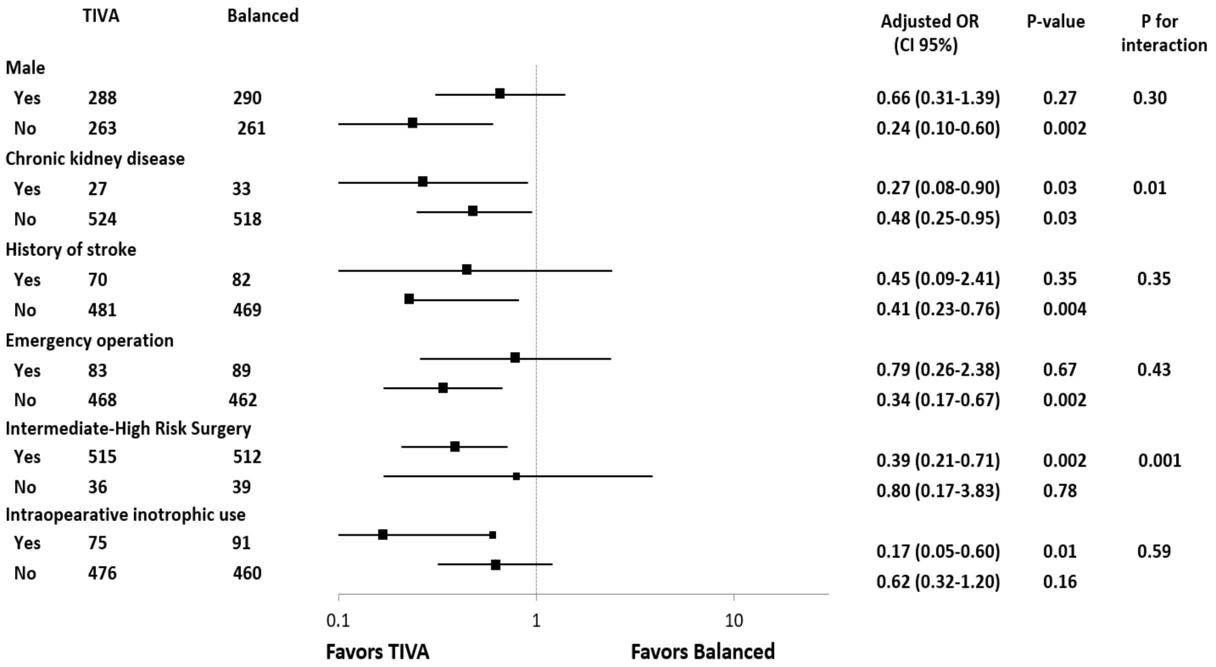

Figure 2. Subgroup analysis for AKI.
